# PD-L1 and MHC-I expression in 19 human tumor cell lines and modulation by interferon-gamma treatment

**DOI:** 10.1186/2051-1426-2-S3-P102

**Published:** 2014-11-06

**Authors:** Italia Grenga, Renee N Donahue, Lauren Lepone, Jessa Bame, Jeffrey Schlom, Benedetto Farsaci

**Affiliations:** 1 Laboratory of Tumor Immunology and Biology, CCR, NCI, NIH, Bethesda, MD, USA; 2 NCI/CCR/LTIB, Bethesda, MD, USA

## Background

The aim of this study was to analyze the expression of PD-L1 and MHC-I in 19 human tumor cell lines and changes after interferon gamma (IFN-γ) treatment, in order to evaluate the potentiality of combining anti-PD-L1 antibody with other immunotherapies.

## Methods

Nineteen human tumor cell lines were cultured according with ATCC guidelines: 5 colon (Caco-2, SW620, SW480, Colo-205 and HT-29), 4 ovarian (OV-17, OVCAR-3, ES-2, SKOV-3), 3 breast (MDA-MB-231, MCF-7, ZR-75), 3 lung (H441, H1703, H460), 2 prostate (LnCap and PC-3), and 2 pancreatic (CFPAC-1 and ASPC-1). Cells were analyzed by flow-cytometry for PD-L1 (clone 29E.2A3) and MHC-I expression. The surface expression of PD-L1 was considered as low, medium, or high based on the percentage of positive cells (80%, respectively). Cells were also analyzed for PD-L1 mRNA expression by RT-PCR. Experiments were performed with or without IFN-γ pre-treatment (10 ng/ml, 24 hours).

## Results

The expression of PD-L1 was as follows. Low: 4/5 colon (SW620, SW480, Colo-205 and HT-29), 1/4 ovarian (OVCAR-3), 2/3 breast (ZR-75, MCF-7), and 1/2 pancreatic (ASPC-1). Medium: 1/5 colon (Caco-2), 2/4 ovarian (OV-17, SKOV-3), 2/3 lung (H460, H1703), and 1/2 prostate (LnCap). High: 1/4 ovarian (ES-2), 1/3 lung (H441), and 1/2 prostate (PC-3), 1/3 breast (MDA-MB-231), and 1/2 pancreatic (ASPC-1). After IFN-γ pre-treatment, 14/19 cell lines showed a >50% increase of PD-L1 and 14/19 a >50% increase of MHC-I (either percentage positive or MFI). In 13/19 cell lines both markers increased. IFN-γ pre-treatment caused an increase >100% of PD-L1 mRNA expression in 14/19 cell lines. CFPAC-1 (pancreatic) showed an increase of surface PD-L1 without mRNA change; on the opposite, H1703 (lung) showed mRNA increase without changes in surface expression.

## Conclusions

Tumor cells express different percentage of PD-L1 and MHC-I in their surface. In most of the cells analyzed, both molecules are increased by exposure to IFN-γ. Based on these observations, immunotherapies aiming to increase IFN-γ in the tumor microenvironment, such as therapeutic vaccines or T cell adoptive transfer, can facilitate immune recognition of tumor cells by an increase of MHC-I on the surface of tumor cells. On the other hand, the increased PD-L1 expression in the tumor can be an ideal target for anti-PD-L1 antibody treatment.

